# From concepts, theory, and evidence of heterogeneity of treatment effects to methodological approaches: a primer

**DOI:** 10.1186/1471-2288-12-185

**Published:** 2012-12-13

**Authors:** Richard J Willke, Zhiyuan Zheng, Prasun Subedi, Rikard Althin, C Daniel Mullins

**Affiliations:** 1 Pfizer, Inc, 235 E. 42nd Street, New York, NY 10017, USA; 2 University of Maryland School of Pharmacy, 220 Arch Street, 12th Floor, Baltimore, MD, 21201, USA; 3 Pfizer, Inc, 500 Arcola Road, Collegeville, PA, 19426-3982, USA

**Keywords:** Heterogeneity, Risk adjustment, Estimation techniques, Comparative effectiveness research

## Abstract

Implicit in the growing interest in patient-centered outcomes research is a growing need for better evidence regarding how responses to a given intervention or treatment may vary across patients, referred to as heterogeneity of treatment effect (HTE). A variety of methods are available for exploring HTE, each associated with unique strengths and limitations. This paper reviews a selected set of methodological approaches to understanding HTE, focusing largely but not exclusively on their uses with randomized trial data. It is oriented for the “intermediate” outcomes researcher, who may already be familiar with some methods, but would value a systematic overview of both more and less familiar methods with attention to when and why they may be used. Drawing from the biomedical, statistical, epidemiological and econometrics literature, we describe the steps involved in choosing an HTE approach, focusing on whether the intent of the analysis is for exploratory, initial testing, or confirmatory testing purposes. We also map HTE methodological approaches to data considerations as well as the strengths and limitations of each approach. Methods reviewed include formal subgroup analysis, meta-analysis and meta-regression, various types of predictive risk modeling including classification and regression tree analysis, series of n-of-1 trials, latent growth and growth mixture models, quantile regression, and selected non-parametric methods. In addition to an overview of each HTE method, examples and references are provided for further reading.

By guiding the selection of the methods and analysis, this review is meant to better enable outcomes researchers to understand and explore aspects of HTE in the context of patient-centered outcomes research.

## Review

### Background

Recent interest in ‘patient-centered’ outcomes research (PCOR) stems from a growing need for valid and reliable evidence that can be used by stakeholders to make individualized treatment decisions. Currently, patients, physicians and payers often make treatment or payment decisions based upon data representing the average effect of an intervention, as observed from selected pools of patients in a clinical trial setting. Reliance on trial outcomes data for real-world decision making requires an assumption that the study population from which the average was generated accurately represents the individual patient. However, a ‘real’ patient is likely different from the average trial patient in important ways, such as demographic, disease severity, or health behavior characteristics.

In terms of intervention outcomes, these differences could mean that the average effect observed in the study population may bear little resemblance to the real effect observed in the individual patient [1,2]. Growing awareness of this phenomenon – known as heterogeneity of treatment effect (HTE) – has fueled recent discussions regarding how PCOR studies can be designed to better account for HTE, so that the results of such research can guide treatment and insurance coverage decision making. Given a pressing need to achieve better value in health care spending, timely HTE evidence can contribute to both more individualized and more efficient care. While observational studies have and will continue to play an important role in generating PCOR evidence, there is also an opportunity to more consistently incorporate HTE considerations in the design and analysis of randomized clinical trials (RCT).

**Figure 1:**
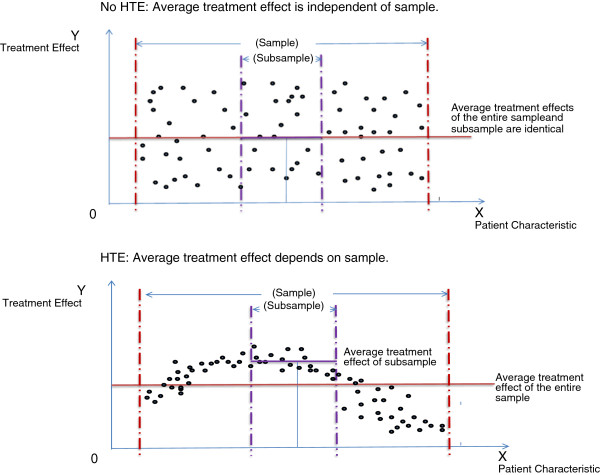
Random Variation in Treatment Effect vs. Heterogeneity of Treatment Effect.

Figure 1 illustrates the impact of HTE on ‘averages’. In the top portion of the Figure, the treatment effect (represented on the Y axis) is randomly distributed and independent of the patient characteristic of interest (the X axis). In this case, the observed average treatment effect is consistent, regardless of whether the entire sample or a subsample similar to the patient is considered. Thus, in this random effects scenario, HTE does not exist. In contrast, the bottom portion of the Figure shows a scenario in which the treatment effect is highly dependent on the distribution of the patient characteristic. Here, a treatment decision based upon the average effect observed in the entire sample would underestimate the average effect in the subsample that is similar to the patient. The treatment decision dilemma regarding whether to expect that the average effect from a trial will predict an individual’s specific treatment effect is not novel; but modern methodological approaches have stimulated renewed efforts to bridge the gap between the two, recognizing that treatment decisions are a function of the evidence informing them – and that different choice(s) may be made when more patient-centered outcomes evidence is available to decision-makers.

Assessment of HTE is becoming more common in the medical literature, often in the form of subgroup analysis within RCTs [3]. Formal, preplanned subgroup analysis of RCT data is certainly one means of analyzing HTE (and is discussed further below), but may not represent the most efficient or appropriate approach to investigating HTE. This is because HTE may be the result of complex interactions or latent factors that can only be unveiled by more elaborate empirical strategies. The purpose of this paper is to outline a set of key considerations for conducting prospective HTE research through a discussion of a number of validated HTE methodological approaches. We systematically address how background prior beliefs (“priors”) and pre-existing evidence can shape a methodological approach to better understand HTE. Aimed at study designers with an “intermediate” level of understanding of both statistics and HTE, this paper provides an overview of select methods for evaluating HTE. Along with a description of each method is guidance for their most appropriate applications. This paper can be used as a starting point for an audience that may need to factor HTE considerations into their research plans, but may be unfamiliar with the full constellation of methods available.

This article is intended to serve as a thorough discussion about how to explore, evaluate and evaluate HTE evidence, a primer of sorts, for researchers in all sectors but particularly those involved in product development requiring RCT’s, who are interested in incorporating HTE considerations into their RCT studies. Because, for a given study, aims and circumstances can vary widely, we have sought to avoid a prescriptive approach to the process of methods selection. Instead, we seek to provide the reader with a general framework, supplemented with sufficient background material that, when combined with examples and references, enable the researcher who is interested in developing their own HTE study with the tools needed to do so.

The HTE methodological approaches discussed below were informed by a literature review of HTE methods and selected to provide a review of a variety of techniques in the medical, statistics, and economics literature. In order to focus on methods useful in product development, they were also selected for their apparent relevance to RCT conduct and analysis. Non-randomized data analyses can inform trial design and real-world comparative effectiveness research (CER) studies, but are also subject to treatment selection biases which can significantly complicate HTE analysis. We considered such issues beyond the scope of this paper and so non-randomized data applications are included to a much lesser degree. Also beyond this paper’s scope is the background science needed to analyze genetically-driven differences, although the HTE methods discussed below may be used in an exploratory manner in that area.

We begin with relatively established approaches such as formal subgroup analysis of clinical trial data, and heterogeneity in meta-analysis of trials. We then discuss more exploratory approaches for HTE, particularly the family of predictive risk modeling approaches, with some detail on classification and regression tree (CART) analysis. This is followed by several approaches more explicitly accounting for intra-individual effects, albeit in quite different ways – latent growth and growth mixture models, and series of “n-of-1” trials - with the latter being an example of how an alternative trial design can be combined with specific methodological approaches to model HTE. Finally, we discuss some HTE methods receiving relatively more attention in the econometrics literature: quantile-based treatment heterogeneity and non-parametric methods testing for HTE. The overview of methods is followed by a discussion of how those with an interest in PCOR and HTE during product development can develop an appropriate research agenda by appropriately matching methods with study questions.

### Subgroup analysis of clinical trial data

Widespread in use but contentious in value, subgroup analysis can assess whether observed clinical trial treatment effects are consistent across all patients, or whether HTE exists across the patient population’s demographic, biologic, or disease characteristics. Subgroup analyses may be pre-specified as part of a trial’s analysis plan; however, reviews of clinical trial reports suggest that subgroup analyses are often employed when a statistically significant effect is not detected in the overall population in an effort to identify a statistical effect of interest [4,5].

Subgroup analysis should be both undertaken and interpreted with caution, especially when not pre-specified [6,7]. One key issue in subgroup analyses is incomparability of the subpopulations of interest, which can arise when the trial’s patient randomization process has not appropriately taken into account the factors that define the subgroup [8]. In cases where subgroup analyses are pre-specified as part of the clinical trial protocol, imbalances may be minimized through appropriate stratification during randomization.

Sample size and power are additional concerns, as most trials are powered to only detect treatment differences on the overall population level. Even in cases where a significant effect within a subgroup is detected, multiplicity is also a concern, as the likelihood of a false-positive response increases significantly with each additional analysis conducted [9]. Established adjustment techniques (such as the Bonferroni, Hochberg, and related methods) can help to adjust for the multiplicity issue. However, depending on the number of analyses conducted, this adjustment, in conjunction with power limitations, may significantly reduce the likelihood of observing a true effect [9]. Clear guidance has been developed regarding the appropriate approaches to subgroup analyses; these overviews provide a clear framework for how subgroup analyses should be undertaken [10,11].

Several studies have demonstrated how spurious results from subgroup analyses can be easily, if inadvertently, generated [7,12,13]. The publication and over-interpretation of these likely false findings only serves to increase skepticism around the potential value of subgroup analyses. Subgroup analyses have greatest value when used to generate new hypotheses that can be appropriately tested in experimental studies that are specifically designed to test these questions and balance potential imbalance, power, and multiplicity issues.

### Meta-analysis

Meta-analysis is a technique that can be used to combine treatment effects across trials and their variations into an aggregated treatment effect with higher statistical power than observed in the individual trials. It can also provide an opportunity to detect HTE by testing for differences in treatment effects across similar RCTs. It is important that the individual treatment effects are similar enough for the pooling to be meaningful. If there are large clinical or methodological differences between the trials, an appropriate judgment may be to not perform a meta-analysis at all.

HTE across studies included in a meta-analysis may exist because of differences in the design or execution of the individual trials (such as randomization methods, patient selection criteria, handling of intermediate outcomes, differences in treatment modalities, etc). Cochran's Q, a technique to detect this type of statistical heterogeneity, is computed as the weighted sum of squared differences between each study's treatment effect and the pooled effects across the studies, and provides an indication of whether inter-trial differences are impacting the observed study result [14]. Similarly, tests such as the I^2^, H^2^, and R^2^ indices developed by Higgins and Thompson are closely related and measure the degree of statistical heterogeneity [15].

A possible source of error in a meta-analysis is publication bias, i.e., the likelihood that a particular trial result is reported depends on the statistical significance and the direction of the result. Trial size might also result in publication bias since larger trials would be less likely to escape publication than smaller, less-known ones. Language and accessibility might be other factors. There are methods of identifying and adjusting for publication bias, e.g. the funnel plot which plots the effect size against the sample size and if no bias is present is shaped as a funnel [16]. The 'trim and fill' method is a non-parametric method of adjusting for publication bias based on the funnel plot [17,18]. Significance tests such as the Egger's test and Begg's test can also be used to identify publication bias [19,20]. Identifying and adjusting for publication bias, in the presence of HTE, has been shown to be difficult when the meta-analysis is not large [21]. As has been noted, however, not all HTE is bad [22]. If the heterogeneity is not a consequence of poor study design it should be welcomed as a possibility to optimize treatment benefits for different patient categories.

HTE can sometimes be minimized by choosing the measure with the smallest variance across trials. Common measures of treatment effect when comparing proportions are: the additive Risk Difference (RD), and the multiplicative Relative Risk (RR) and Odds Ratio (OR). The RR and the OR both have good statistical properties but are less intuitive to interpret than the less statistically efficient RD. It may be the case that one measure, such as the RR, does not vary across subgroups, while the RD does vary, and it likely will if a common RR is applied to different baseline risks [22,23]. In such cases it is important to determine what type of HTE is meaningful for the purpose at hand.

Meta-regression is a variant of meta-analytic technique that allows for a more in-depth understanding of the pooled clinical trial data, by “exploring whether a linear association exists between variables and a comparative treatment effect, along with the direction of that association” [24]. As pointed out by Baker et al, meta-regression should not be undertaken unless there is a sound rationale for the hypothesis that one, or more, covariates vary linearly with the treatment effect (e.g. what effect a one year increase in age has on the treatment effect) [24].

### Predictive risk modeling

A rapidly growing method for identifying potential for HTE is predictive risk modeling, whereby individual patient risk for disease-related events at baseline is differentiated based on observed factors. Most common measures are disease staging criteria, such as those used in COPD or heart failure, as well as more continuous algorithmic measures such as the Framingham risk scores for cardiovascular event risk [25,26]. Genetic variations, such as HER2 for breast cancer or cytochrome p-450 polymorphisms, are other such factors [27].

Initial predictive risk modeling, also known as risk function estimation, is often but not always performed prior to including treatment effects, and can employ a variety of methods. This is an area of extensive current research for more efficient algorithms, given the proliferating sources of individual patient data with large sample sizes, better data on outcomes, and large numbers of potential predictors. Traditional least squares or Cox proportional hazards regression methods are still quite appropriate in many cases and provide relatively more interpretable risk functions, but are typically based on linearity assumptions and may not give the highest scores for predictive metrics. Partial least squares is an extension of least squares methods that can reduce the dimensionality of the predictor space by interposing latent variables, predicted by linear combinations of observable characteristics, as the intermediate predictors of one or more outcomes [28]. Other, less interpretable methods include various types of recursive partitioning, such as random forests, support vector machines, and neural networks [29-32]. Some of these latter methods, particularly support vector machines, have been shown to often have better predictive success than more linear methods, generally at the expense of clarity of the risk mechanisms. Risk function estimation can range from highly exploratory analyses to near meta-analytic model validation, and may be useful at any stage of product development. The better validated the risk mechanism, however, the more it can be used for hypothesis-driven rather than exploratory analyses.

Given a risk function that generates pre-treatment event risk predictions for individual patients, one must choose how to use it with RCT data to evaluate HTE. For categorical risk predictions, methods such as subgroup interactions or stratified treatment analyses can be used, subject to the considerations discussed above. Continuous risk predictions can be interacted with the treatment response in a regression format, but questions about the nature of the interaction – linear, quadratic, logistic, etc. – must be managed. Some other techniques have been proposed as well. For example, Ioannidis and Lau propose dividing patients into quartiles based on predicted risks and analyzing accordingly [33]. Lazar et al propose a technique they term “subpopulation treatment effect pattern plot” that evaluates the effect of risk on treatment outcomes in a continuous, non-parametric method, using moving averages over successively higher risk groups [34]. Crown describes a regression-based decomposition method that is useful in parsing out risk factor effects in non-RCT data [35]. With such continuous risk-treatment interactions, if subgroup-determining breakpoints are subsequently needed for decision-making, one approach is post-hoc application of clinically meaningful treatment effects.

### Classification and regression tree (CART) analysis

A decision-tree based technique, the Classification and Regression Tree (CART) approach considers how variation observed in a given response variable (continuous or categorical) can be understood through a systematic deconstruction of the overall study population into subgroups, using explanatory variables of interest [36]. In the context of the various statistical tools that can be used to understand HTE, CART is a simple approach best suited for early-stage, exploratory analyses. CART’s relative simplicity can be powerful in helping the researcher understand basic relationships between variables of interest, and thus identify potential subgroups for more advanced analyses.

The key to CART is its ‘systematic’ approach to the development of the subgroups, which are constructed sequentially through repeated, binary splits of the population of interest, one explanatory variable at a time. In other words, each ‘parent’ group is divided into two ‘child’ groups, with the objective of creating increasingly homogeneous subgroups. The process is repeated and the subgroups are then further split, until no additional variables are available for further subgroup development. The resulting tree structure is oftentimes overgrown, but additional techniques are used to ‘trim’ the tree to a point at which its predictive power is balanced against issues of over-fitting. Because the CART approach does not make assumptions regarding the distribution of the dependent variable, it can be used in situations where other multivariate modeling techniques often used for exploratory predictive risk modeling would not be appropriate – namely in situations where data are not normally distributed.

CART analyses are useful in situations where there is some evidence to suggest that HTE exists, but the subgroups defining the heterogeneous response are not well understood [36]. CART allows for an exploration of response in a myriad of complex subpopulations, and more recently developed ensemble methods (such as Bayesian Additive Regression Trees) allow for more robust analyses through the combination of multiple CART analyses [37,38].

### Latent growth and growth mixture modeling

Latent growth modeling (LGM) is a structural equation modeling technique that captures inter-individual differences in longitudinal change corresponding to a particular treatment. In LGM, patients’ different timing patterns of the treatment effects are the underlying sources of HTE. Not only does LGM distinguish patients who do or do not respond, it also examines whether the patient responds quickly or slowly, and if they have temporary or durable responses. The heterogeneous individual growth trajectories are estimated from intra-individual changes over time by examining common population parameters, i.e., slopes, intercepts, and error variances. For example, each individual has unique initial status (intercept) and response rate (slope) during a specific time interval. The variances of all individuals’ baseline measures (intercepts) and changes (slopes) in health outcomes represent the degree of HTE. The HTE of individual growth curves identified in LGM can also be attributed to observed predictors, including both fixed and time varying covariates. Duncan & Duncan provide a non-technical introduction to LGM [39]. Stull applies LGM to a clinical trial and argues that LGM gives rise to better parameter estimates than the traditional regression-based approach and that LGM can explain a larger proportion of variance [40]. LGM is also closely related to multilevel modeling [41,42].

However, the assumption that all individuals are from the same population in LGM is too restrictive in some research scenarios. If the HTE is due to observed demographic variables, such as age, gender, and marital status, one may utilize multiple-group LGM. Despite its successful applications for modeling longitudinal change, there may be multiple subpopulations with unobserved heterogeneities. Growth mixture modeling (GMM), built upon LGM, allows the identification and prediction of unobserved subpopulations in longitudinal data analysis. Each unobserved subpopulation may constitute its own latent class and behave differently than individuals in other latent classes. Within each latent class, there are also different trajectories across individuals; however, different latent classes don’t share common population parameters. For example, Wang and Bodner use a simulated dataset to study retirees’ psychological well-being change trajectory when multiple unknown subpopulations exist [43]. They add another layer (the latent class variable) on the LGM framework so that the unobserved latent classes can be inferred from the data. Moreover, the covariates in GMM are designed to affect growth factors distinctly across different latent classes. Therefore, there are two types of HTE: 1) the latent class variable in GMM divides individuals into groups with different growth curves; and 2) coefficient estimates vary across latent classes. Donald Stull et al apply GMM to identify and characterize differential responders to treatment for COPD [44]. In comparison of LGM and GMM focusing on longitudinal data, Luke & Muthen discuss factor mixture modeling as a method for cross-sectional studies when heterogeneous populations arise in a similar fashion as in GMM [45].

Wang and Bodner and Jung & Wickrama provide intuitive introductions to GMM [43,46]. Both point out that the precision of GMM depends on the number of predictors included in the model. Moreover, the optimal number of latent classes needs to be determined. In the two-step approach they discuss, different criteria, e.g. Akaike Information Criterion and Bayesian Information Criterion can be applied for this purpose, but they are sensitive to sample sizes. GMM also comes with potential computational burden, and may result in non-convergence or local solutions.

### Series of n of 1 trials

Combined (aka, “series of”) n-of-1 trial data provide a unique way to identify HTE. An n-of-1 trial is a repeated crossover trial for a single patient, which randomly assigns the patient to one treatment vs. another for a given time period, after which the patient is re-randomized to treatment for the next time period, usually repeated for 4-6 time periods. Such trials are most feasibly done in chronic conditions, where little or no washout period is needed between treatments and treatment effects are identifiable in the short-term, such as pain or reliable surrogate markers [47-49].

**Figure 2:**
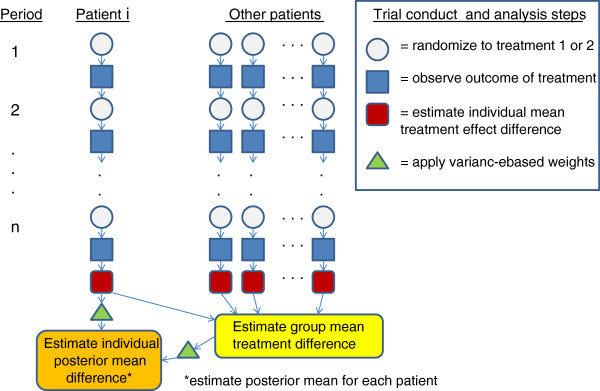
Series of N-of-1 Trials, Conduct and Analysis Steps.

Combining data from identical n-of-1 trials across a set of patients allows for a powerful statistical analysis which can control for patient fixed or random effects as well as covariate, center, or sequence effects. These combined trials are often analyzed within a Bayesian context using shrinkage estimators that combine individual and group mean treatment effects to create a “posterior” individual mean treatment effect estimate which is a form of inverse variance-weighted average of the individual and group effects. These trial conduct and analysis steps are illustrated in Figure 2. While such trials are typically more expensive than standard RCTs on a per-patient basis, the statistically efficient individual-as-own-control design allows for much smaller sample sizes, often less than 100 patients, and creates individual treatment effect estimates that are not possible in a non-crossover design. For the individual patient, the treatment effect can be re-estimated after each time period, and the trial stopped at any point when the more effective treatment is identified with reasonable statistical certainty [50].

Zucker et al furnish a good example of the information provided by analysis of combined n-of-1 trials [50]. N-of-1 trials were conducted for 23 fibromyalgia patients, comparing amytriptyline with placebo for up to six time periods. The overall mean difference in the disease evaluation score was significantly positive but slightly below a level considered clinically significant. However, the posterior means of the treatment effect for 10 patients were greater than the clinically meaningful threshold, a signal that the treatment was beneficial to a subset of the patients.

Combined n-of-1 trials could be used for early exploratory testing for HTE, or for later-phase, more focused testing of comparative effectiveness or cost-effectiveness [51,52]. While not currently well-accepted for regulatory purposes, in today’s highly competitive environment for chronic treatments, n-of-1 trials could provide HTE information useful in creating phase 3 trial designs that may lead to evidence more clearly differentiating new treatments.

### Quantile regression

Quantile regression provides additional distributional information about the central tendency and statistical dispersion of the treatment effect in a population, which is not normally revealed by the conventional mean estimation in RCTs. For example, patients with different comorbidity scores may respond differently to a treatment. Quantile regression has the ability to reveal HTE according to the ranking of patients’ comorbidity scores or some other relevant covariate by which patients may be ranked. Therefore, in an attempt to inform patient-centered care, quantile regression provides more information on the distribution of the treatment effect than typical conditional mean treatment effect estimation. The quantile treatment effect (QTE) characterizes the heterogeneous treatment effect on individuals and groups across various positions in the distributions of different outcomes of interest. This unique feature has given quantile regression analysis substantial attention and has been employed across a wide range of applications, particularly when evaluating the economic effects of welfare reform [53-55].

**Figure 3:**
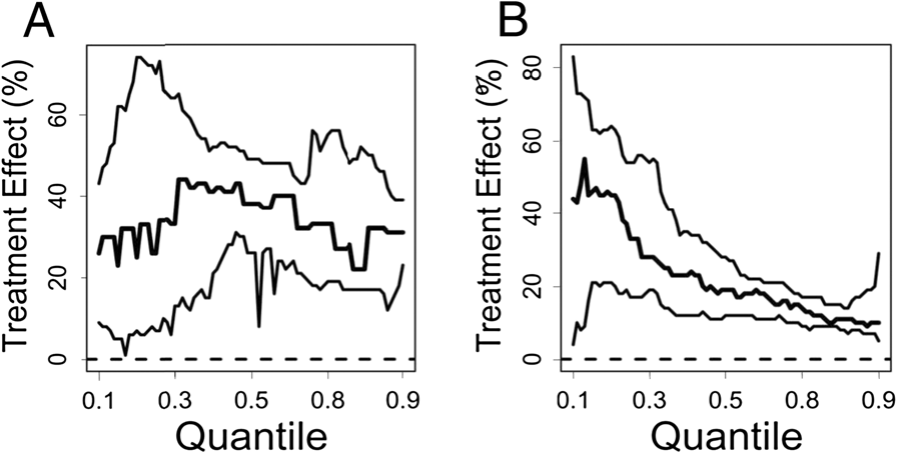
**Quantile Regression Estimation of Treatment Effects. **In the above Figure, quantile regression is used to estimate treatment effects across a range of survival time quantiles (*τ* = 0.10, …, 0.90). For a given quantile *τ* (horizontal axis), the vertical axis represents the percent increase in survivorship of Mediterranean fruit flies associated with an experimental treatment. In part (A), results for the *PappA*(−/−) treatment are shown, and in part (B), results for the *bIrs2*(+/−) treatment are shown. In each plot, the middle line represents the calculated effect of experimental treatments at each survival time quantile, while the upper and lower lines outline a 95% confidence region. It is clear that in Part A, treatment effects don’t change with respect to the specific quantile. However, in Part B, the treatment effects decrease as the quantile increases.Reference: Swindell (2009) [57]. Reproduced with permission.

One caveat of applying quantile regression in clinical trials for examining HTE is that QTE doesn’t demonstrate the treatment effect for a given patient. Instead, quantile regression focuses on the treatment effect among subjects within the *q*th quantile, such as those who are exactly at the top 10^th^ percent in terms of blood pressure or a depression score for some covariate of interest, for example, comorbidity score. It is not uncommon for the *q*th quantiles to be two different sets of patients before and after the treatment. For this reason, we have to assume that these two groups of patients are homogeneous if they were in the same quantiles. For example, Bitler et al [56] make the above homogeneous distributional assumption and study HTE of welfare reforms by using the experimental data from Connecticut’s Jobs First waiver. The QTE is measured as the difference between the inverse cumulative density functions of the treatment group and the control group. Another example, based on survival time quantiles, is shown in Figure 3[57].

The literature on HTE of the impact of welfare reform has focused on mean treatment effects across demographic subgroups. This leads us to assume that the potential HTE results from observed differences in some demographic characteristics. It is possible that the statistical significance of observed HTE from subgroup analysis is due to some outliers in the dataset, especially when the number of patients in a subgroup is relatively small. Quantile regression has the advantage of being robust to outliers. In a RCT where outliers are a potential issue, QTE will certainly have the superior performance compared with subgroup analysis and provide more convincing evidence for HTE.

### Nonparametric methods

**Figure 4:**
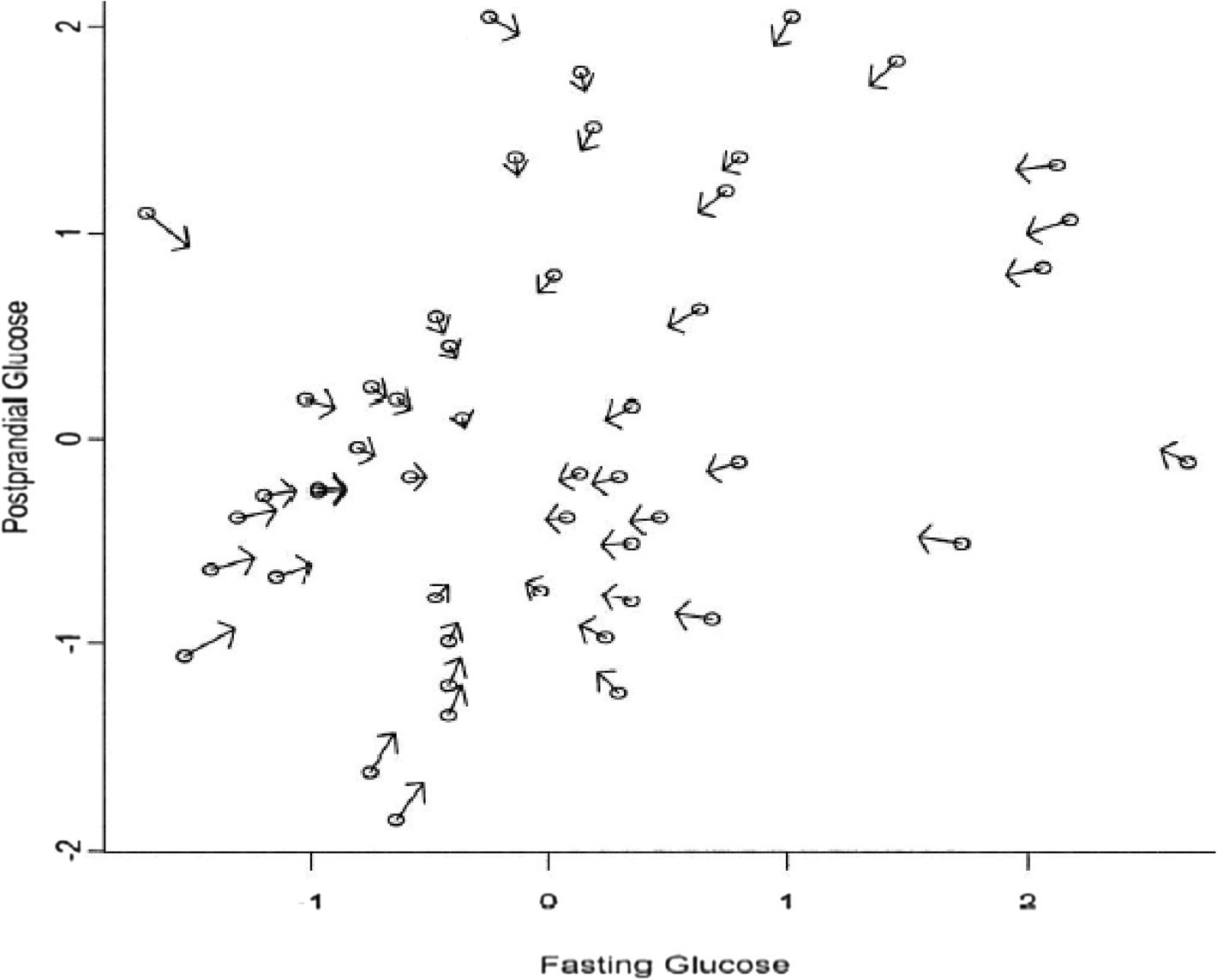
**Nonparametric Regression Estimation of Treatment Effects. **Blood-sugar measurements are a common tool in diabetes testing. In a glucose-tolerance test, the glucose level in blood is measured after a period of fasting (fasting-glucose measurement) and again 1 h after giving the subject a defined dose of glucose (postprandial glucose measurement). Pregnant women are prone to develop subclinical or manifest diabetes, and establishing the distribution of blood-glucose levels after a period of fasting and after a dose of glucose is therefore of interest.The above Figure shows that bivariate nonparametric regression to the mean for glucose measurements for 52 women, with repeated measurements over three pregnancies. Circles are observed sample means obtained from the three repetitions of the standardized values of (fasting glucose, postprandial glucose). Arrows point from observed to predicted values. It is clear that different women have different treatment effects represented by the directions of the arrows.Reference: Müller et al (2003) [58]. Reproduced with permission.

Nonparametric methods have a variety of approaches and advantages for dealing with HTE in RCTs. Different nonparametric methods, such as kernel smoothing methods and series methods, can be used to generate test statistics for examining the presence of HTE. A kernel method is a weighting scheme based on a kernel function (e.g. uniform, Epanechnikov, and Gaussian). When evaluating the treatment effect of a patient in RCTs, the kernel method assigns larger weights to those observations with similar covariates. This is done because it is assumed that patients with similar covariates provide more relevant data on predicted treatment response. For patients who have significantly different backgrounds (demographically, clinically, and contextually speaking), kernel smoothing methods still utilize information from highly divergent patients when estimating a particular patient’s treatment effect; however, lower weights are given to patients who are very different. Additionally, kernel methods require choosing a set of smoothing parameters to group patients according to their relative degree of similarities. Figure 4 provides an illustration of how this smoothing can modify individual estimates [58]. However, the drawback is that the corresponding proposed test statistics might be sensitive with respect to the chosen bandwidths, which makes the interpretation of results confusing. Series methods use approximating functions (splines or power series of the explanatory variables) to construct test statistics. Compared to kernel smoothing methods, series methods normally have the advantage of computational convenience; however, the precision of test statistics depends on the number of terms selected in the series.

Nonparametric test statistics have been proposed in the literature as powerful tools to identify potential HTE. Crump et al [59] propose two test statistics for experimental evaluations of welfare reforms by using the power series method to test whether the average effects of a binary treatment are zero or constant over different subpopulations defined by covariates. Lee proposes a kernel smoothed nonparametric test for heterogeneity of conditional treatment effects when covariates are continuous and the outcome variable is randomly censored [60].

The significance of utilizing nonparametric models lies in the less restrictive assumptions (i.e., differentiability and moment conditions) imposed in comparison with the functional form assumptions of their parametric counterparts. More often than not, the structure in parametric models implicitly assumes a homogeneous treatment effect. Therefore, some nonparametric regression frameworks are flexible in their designs so that they permit HTE across individual patients. Rather than providing a p-value for the existence of HTE, the nonparametric regression frameworks may present treatment effects that vary among patients, from which the distribution of the response to a treatment is observable. The underlying hypothesis is that differential treatment response can be explained by differences in patients’ demographic characteristics, clinical variables, and contextual variables. Frolich considers a local likelihood logit model for binary dependent variables [61]. The proposed estimator combines the parametric logit function with the nonparametric kernel smoothing framework. The HTE is identified by looking at varying conditional means and marginal effects for particular changes in the observable covariates.

## Discussion and conclusion

Two factors motivated the generation of this primer. First was a growing recognition of the interest in and need for more granular and patient-centric data with which individualized treatment decisions could potentially be made. Second was a realization that there were no unifying guiding principles for those researchers who might be interested in exploring HTE as part of a PCOR agenda.

**Figure 5:**
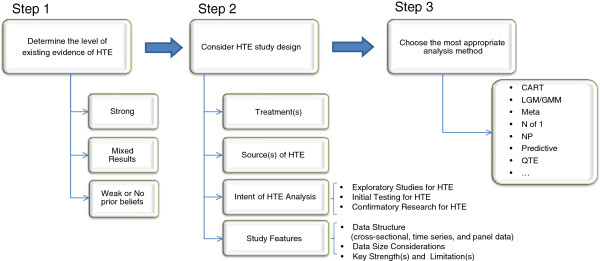
Decision Process for Choosing a Method for HTE.

Figure 5 represents our attempt to build a general framework around these principles, outlining some of the considerations that can inform the design of HTE studies for medical products – particularly pharmaceutical but potentially others such as device or surgical – at different phases of development. An important initial consideration in the selection of a HTE methodology is the level of prior evidence regarding the existence and nature of HTE for the treatment(s) in question. The level of evidence may vary widely – for example, early stage studies may be informed only by biological theory or by evidence from similar existing treatments, while post-marketing studies may be informed by original or subsequent analysis of the phase 3 data, by actual case studies, or by real world comparative effectiveness studies.

Once the level of prior information is established, a second consideration relates to the development of HTE study objectives. Key elements include the treatments to be studied as well as the prior evidence about the nature and sources of HTE, which may be population-based or treatment-based or both. These will inform the intent of the HTE investigation, whether it is to be largely exploratory or testing specific hypotheses. Early stage studies with little prior evidence may need to be more exploratory in nature. Subsequent phase 3 studies may need to determine the most appropriate doses and populations for initial labeling and may be designed to test very specific hypotheses already formed by such studies.

Another key element is the nature of the data available for the study. Existing trial or real-world data may be sufficient for the intent of the study, or may be the only study options; otherwise, new data collection may be part of the study design. Finally, the researcher must choose the specific method(s) to test for HTE in the study data. Table 1 provides the most concise representation of our primer’s intent by arraying key considerations related to the intent of potential data and methodological needs against each of the seven approaches discussed above, providing context for when a given approach might prove most useful. As is often the case in overviews such as this, it is difficult to declare a single method or two as being the most appropriate for all (or even most) situations in which HTE is suspected to exist and warrants further exploration. The complex construct of HTE is made more challenging by analytic constraints. We have sought to provide clear and novel guidance in both the text and the Table as to what some of the tradeoffs are when, for example, selecting between a post-hoc analysis as compared to an n-of-1 study. As might be expected, we observe that when the intent of an HTE study moves from exploratory to confirmatory, so do the technical challenges associated with a given methodological approach – in terms of more complex dataset structures, assumptions related to distributions, or sample size considerations. It seems safe to say then that the appropriate selection of a statistical framework for a given HTE research question should seek to weigh the level of existing evidence of HTE against these intent and methodological burden factors, and that in any reporting of HTE study results, study findings should be contextualized against the approaches used to derive them. In addition, for the benefit of those who would like to explore specific analytical tools for these approaches, we provide notes on some available estimation routines in well-known software in an Appendix.

**Table 1:** Features of selected approaches to analysis of HTE

	***Meta-analysis***	***CART***	***N of 1 trials***	LGM/GMM*	QTE**	***Nonparametric***	***Predictive risk models***
*Intent of HTE Analysis*	*· Exploratory and confirmatory*	*· Exploratory*	*· Exploratory and initial testing*	*· Exploratory, initial testing, and confirmatory*	*· Exploratory, initial testing, and confirmatory*	*· Exploratory and confirmatory*	*· Initial testing and confirmatory*
*Data Structure*	*· Trial summary results, possibly with subgroup results*	*· Panel or cross-section*	*· Repeated measures for a single patient: time series*	*· Time series and panel*	*· Panel and cross-sectional*	*· Panel, time series, and cross-sectional*	*· Panel or cross-sectional*
*Data Size Consideration*	*· Advantage of combining small sample sizes*	*· Large sample sizes*	*· Small sample sizes*	*· LGM: small to large sample sizes*	*· Moderate to large sample sizes*	*· Large sample sizes*	*· Sample sizes depends on specific risk function*
				*· GMM: Large sample sizes*			
*Key Strength(s)*	*· Increase statistical power by pooling of results*	*· Does not require assumptions around normality of distribution*	*· Patient is own control*	*· Accounting for unobserved characteristics*	*· Robust to outcome outliers*	*· No functional form assumptions*	*· Multivariate approach to identifying risk factors or HTE*
			*· Estimates patient-specific effects*				
					*· Heterogeneous response across quantiles*	*· Flexible regressions*	
				*·Heterogeneous response across time*			
	*· Possible to identify HTE across trials*	*· Can utilize different types of response variables*					
	*· Possibility to measure and explain covariate's effect on treatment effect*						
*Key Limitation(s)*	*· Included studies need to be similar enough to be meaningful*	*· Fairly sensitive to changes in underlying data*	*· Requires de novo study*	*· Criteria for optimization solutions not clear*	*· Treatment effect designed for a quantile, not a specific patient*	*·Computationally demanding*	*· May be more or less interpretable or useful clinically*
			*· Not applicable to all conditions or treatments*			*· Smoothing parameters required for kernel methods*	
		*· May not fully identify additive impacts of multiple variables*					
	*· Assumed distribution*						
	*· Selection bias*						

* LGM/GMM: Latent growth modeling/Growth mixture modeling.

**QTE: Quantile treatment effect.

The importance of ‘context’ for HTE studies goes beyond just methodological concerns. The recent and growing interest in patient-centered treatment means that HTE studies are increasingly likely to be used in clinical decision-making. However, the hope that HTE evidence can serve to significantly improve patient outcomes needs to be balanced against questions regarding the reliability of the scientific methodology used to identify the HTE in question, the weight of what is already understood about conditions in which HTE studies are developed, and new concerns that may arise as additional HTE evidence is generated.

An example of such a concern is whether it is sufficient to be able to detect that a given population responds differentially to a treatment. What if this differential response is novel, was not previously detected in prior attempts at HTE investigation, but was only discovered using a relatively new statistical technique? Should those patients who reflect the differential response population be treated differently – and if so, how?

The key question facing researchers and policy makers is as follows: what level of evidence is required before treatment paradigms may change on the basis of HTE data, and how do we understand and accept the validity of this evidence in a landscape where new tools for detection are consistently being developed? This question is only likely to become more urgent as increased availability of electronic data sources yields more and more research that could profoundly impact clinical treatment paradigms. While there is great hope that recent efforts like those being undertaken by the Patient Centered Outcomes Research Institute will result in the development of better evidence and improved decision-making ability for all stakeholders, significant work is needed to standardize and build consensus around which methods are most appropriate to be used to generate this evidence.

Despite the high levels of enthusiasm and funding directed towards evidence generation, key questions regarding dissemination and assimilation of evidence into clinical practice remain. In the case and context of HTE studies, it will be crucial to further understand what types of analyses are most likely to impact clinical decision-making behavior. By contextualizing various existing HTE methods that could potentially be used against a novel framework, which highlights both prior evidence as well as other considerations related to elements of study design, this primer sought to fill what seems to be an increasingly important gap in the outcomes research literature.

## Appendix – notes on estimation routines

Standard meta-analysis like fixed and random effect models, and tests of heterogeneity, together with various plots and summaries, can be found in the R-package rmeta (http://cran.r-project.org/web/packages/rmeta). Logistic regression and survival model routines, both basic approaches to predictive modeling, are found in all major statistical packages (e.g., SAS (Proc Logistic, …), Stata (logistic or logit, stcox, etc.). Routines for calculating empirical Bayes shrinkage estimates for n-of-1 trials are available in S-Plus, with more general Bayesian approaches available in WinBUGS or in R with R2WinBUGS. Basic quantile regressions can be estimated in Stata with the command qreg or in SAS using Proc Quantreg, although some additional programming is needed to generate the full range of quantile estimates. The linear regression decomposition approach can be implemented in Stata with commands decomp, decompose, and Oaxaca. For non-parametric approaches, R offers many available routines, which are well-documented at http://cran.r-project.org/web/packages/np/vignettes/np.pdf. In SAS, Proc NLMIXED and Proc TRAJ are available for the estimation of LGM/GMM; in Stata LGM is handled within the sem command.

## Abbreviations

HTE: Heterogeneity of treatment effect; PCOR: Patient-centered outcomes research; RCT: Randomized clinical trial; CER: Comparative effectiveness research; CART: Classification and regression tree; RD: Risk difference; RR: Risk ratio; OR: Odds ratio; LGM: Latent growth mixture; GMM: Growth mixture modeling; QTE: Quantile treatment effect.

## Competing interests

C. Daniel Mullins, PhD has served as a consultant and scientific advisor to Pfizer Inc and has received consulting income and research grants from Pfizer Inc. Zhiyuan Zheng, PhD served as a consultant to Pfizer Inc in development of this manuscript. Richard Willke, Prasun Subedi and Rikard Althin are employees of Pfizer Inc. These affiliations are not felt to have created any relevant competing interests given the nature of this manuscript.

## Authors’ contributions

All authors made substantial contributions to the conceptualization and writing of this manuscript. The HTE topics included and their appropriate uses were decided on during joint discussions among the co-authors, and each co-author was responsible for writing at least one major section of the manuscript. All authors read and approved the final manuscript.

## Authors’ information

The authors are health economists by training who work in the field of health economics and outcomes research with a focus on pharmaceuticals. The authors have observed the variety of HTE study methods becoming popular in different disciplines, and have taken a particular interest in how different methods can best contribute to the evidence regarding the optimal use of new treatments among different individuals.

## Pre-publication history

The pre-publication history for this paper can be accessed here:

http://www.biomedcentral.com/1471-2288/12/185/prepub
